# Research on the Structure of Peanut Allergen Protein Ara h1 Based on Aquaphotomics

**DOI:** 10.3389/fnut.2021.696355

**Published:** 2021-06-18

**Authors:** Mengqi Zhang, Liang Liu, Cui Yang, Zhongyu Sun, Xiuhua Xu, Lian Li, Hengchang Zang

**Affiliations:** ^1^School of Pharmaceutical Sciences, Cheeloo College of Medicine, Shandong University, Jinan, China; ^2^Research Institute Pharmacy and Medical Science, University of South Australia, Adelaide, SA, Australia; ^3^Key Laboratory of Chemical Biology, Ministry of Education, Shandong University, Jinan, China; ^4^National Medical Products Administration Key Laboratory for Technology Research and Evaluation of Drug Products, Shandong University, Jinan, China; ^5^National Glycoengineering Research Center, Shandong University, Jinan, China

**Keywords:** peanut allergen protein Ara h1, near infrared spectroscopy, aquaphotomics, protein structure, hydrophobicity

## Abstract

Peanut allergy is becoming a life-threatening disease that could induce severe allergic reactions in modern society, especially for children. The most promising method applied for deallergization is heating pretreatment. However, the mechanism from the view of spectroscopy has not been illustrated. In this study, near-infrared spectroscopy (NIRS) combined with aquaphotomics was introduced to help us understand the detailed structural changes information during the heating process. First, near-infrared (NIR) spectra of Ara h1 were acquired from 25 to 80°C. Then, aquaphotomics processing tools including principal component analysis (PCA), continuous wavelet transform (CWT), and two-dimensional correlation spectroscopy (2D-COS) were utilized for better understanding the thermodynamic changes, secondary structure, and the hydrogen bond network of Ara h1. The results indicated that about 55°C could be a key temperature, which was the structural change point. During the heating process, the hydrogen bond network was destroyed, free water was increased, and the content of protein secondary structure was changed. Moreover, it could reveal the interaction between the water structure and Ara h1 from the perspective of water molecules, and explain the effect of temperature on the Ara h1 structure and hydrogen-bonding system. Thus, this study described a new way to explore the thermodynamic properties of Ara h1 from the perspective of spectroscopy and laid a theoretical foundation for the application of temperature-desensitized protein products.

## Introduction

Peanut (1), also known as *Arachis hypogaea* Linn, is an annual leguminous plant, which is an important source of plant protein. It has multi-functions such as lowering blood sugar, preventing cardiovascular and cerebrovascular diseases, and protecting the spleen and stomach (2, 3). However, the peanut is one of the most severe food allergies in the world. Symptoms can be triggered by tiny amounts of allergens manifesting as severe anaphylaxis (4). And the severity of allergic reactions cannot be determined by the eliciting dose (5). Peanuts and nuts are the most common allergens, with a fatality rate of 87%, and some serious allergic reactions are caused by milk and seafood (6). It is reported that peanut allergy accounts for 59% of the total number of food allergies, and affects approximately three in every 100 children (7). Structural changes of the peanut allergens alter its allergenic potential, including the IgE binding capacity and T-cell stimulation. Thermal processing, such as roasting, increases peanut allergenicity by forming higher order of protein structures and reducing the solubility. On the contrary, extended boiling reduces peanut allergen IgE binding capacity, but does not affect T-cell reactivity (8). Therefore, it is necessary to investigate and elucidate the structural transform information of peanut allergens caused by heating to help us understand the biological system.

A total of 11 types of peanut allergen proteins have been approved by the International Federation of Immunization Nomenclature Subcommittee until now (9), and Ara h1 is the most abundant peanut allergen in peanuts with strong sensitization and a relative molecular mass of 63.5K Da (10, 11). Xu et al. (12) investigated the Ara h1 secondary structure with the help of circular dichroism (CD) and fluorescence spectroscopy. The results indicated that after high-temperature heat treatment, the content of β-sheet and random structure in Ara h1 increased, while the α-helical content reduced. The hydrophobicity of the protein-enhanced and its sensitization ability decreased significantly (12). However, there is a lack of in-depth analysis of its thermodynamic changes, secondary structure, and hydrogen bonding system.

Near-infrared spectroscopy (NIRS) is a powerful, non-invasive, and fast analytical technique (13). NIRS combines with aquaphotomics, as an emerging label-free technology, has attracted much attention in protein structure characterization (14, 15). Aquaphotomics was proposed by Tsenkova (16), which was developed mainly based on NIRS. The water spectral information in the system could be obtained under the disturbance of external factors, which then could be applied to get useful information of the solute reflected by the “mirror” of water structure transform (17). The water here could be applied as a probe to carry out the qualitative and quantitative analysis. Dong et al. (18) utilized aquaphotomics to investigate the structural changes of water solvation shells around human serum albumin (HSA), and the results showed that a more ordered hydrogen-bonded water network was formed around HSA with the increase of HSA concentration. At the same time, aquaphotomics could also be used for diseases or abnormal conditions diagnosis. Jinendra et al. (19) found that aquaphotomics was a suitable tool used for the diagnosis of the soybean mosaic disease incubation period. As for the quantitative analysis, a low methanol concentration predictive model was established by using NIRS combined with aquaphotomics, which could be used to monitor the fermentation process (20), and aquaphotomics could sensitively reflect the solvation, water molecule types, and hydrogen bond changes (21).

In this study, the Ara h1 heating process was focused on and near-infrared (NIR) spectra were collected. Water was utilized as a label-free tool to characterize the structure and the changes of water to reflect the structural changes of Ara h1 with temperature used as a perturbation. Through this study, we would like to establish a new method that could elucidate the peanut allergen protein in the view of spectroscopy. In addition, the knowledge produced from this study would help and provide a theoretical basis for the rational processing of peanuts.

## Materials and Methods

### Materials

Matrix-F near-infrared spectrometer (Bruker, Germany) equipped with a temperature controlling device (qpod2e, Quantum Northwest, USA) was used to acquire the NIR spectra. The microplate reader (Biotek, Vermont, USA) was introduced to determine the Ara h1 content. The water used here was deionized water which reached a resistivity of 18.2 MΩ. cm^−1^ (at 25°C) from Milli-Q ultrapure water machine (Millipore, Massachusetts, USA). All data were processed by MATLAB 2019a (Mathworks, Massachusetts, USA).

**Figure 1:**
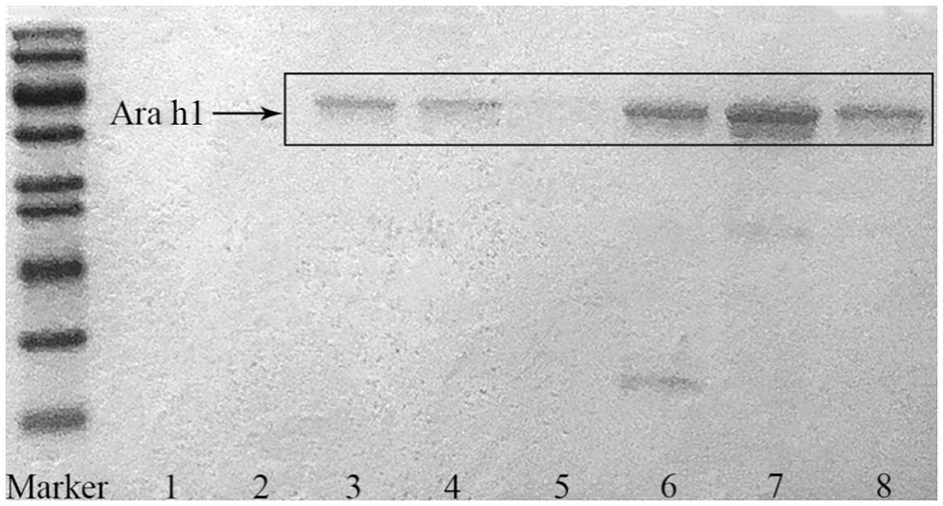
SDS-PAGE of the purified Ara h1.

Ara h1 was separated and purified by acetone degreasing, anion exchange column chromatography, gel column chromatography, and finally prepared by freeze-drying. The purity of Ara h1 was determined by sodium dodecyl sulfate-polyacrylamide gel electropheresis (SDS-PAGE) (the results were shown in Figure 1). Ara h1 was dissolved into the deionized water (20 times diluted) and the Ara h1 content was determined by BCA protein assay kit (Shanghai Biyuntian Biotechnology Co., Ltd.).

### Methods

#### Spectra Collection

The Ara h1 transmission NIR spectra were acquired by Matrix-F near-infrared spectrometer. The wavenumber range was from 12,000 to 4,000 cm^−1^. To apply the aquaphotomics methods, the wavenumber was transformed into wavelength. Therefore, the wavelength in this study was from 833 to 2,500 nm. The resolution was 8 cm^−1^, the optical path was 1 mm, and the number of scans was 64 times. Air was used as the background spectrum, and the Ara h1 spectra were collected from 25 to 80°C with the increment of 5°C. All samples were collected three times at each temperature and the averaged spectra were taken for further analysis.

#### Aquaphotomics Analysis

The collected spectra were processed by multiplicative scatter correction (MSC), and then water matrix coordinates (WAMACS) were calculated following the aquagram used for visual characterization (22, 23). The calculation method was shown in the Formula (1) (24). About 12 different WAMACS were identified to characterize the changes of water structures under temperature disturbances, clarifying the changes of the protein structure based on the spectral differences.

(1) $$\begin{array}{l} A_{\lambda }^{\prime }=\left(A_{\lambda }-\mu_{\lambda }\right)/\sigma_{\lambda } \end{array}$$

In the Formula (1), $A_{\lambda }^{\prime }$, the value on aquagram; *A*_λ_, the absorbance value after MSC treatment; μ_λ_, the average absorbance value at a given wavelength after MSC treatment; σ_λ_, the SD of all spectra at a given wavelength after MSC treatment.

#### Principal component analysis

Principal component analysis (PCA) is extended to random vectors from Hotelling (25), the primary purpose aims to reduce the dimensionality of the data matrix, eliminate overlapping information, and form a new spectral matrix. PCA decomposes the spectral array X (*n* × m) into the sum of the outer products of m vectors, which is expressed by Formula (2) (26).

(2) $$\begin{array}{l} \text{X}=t_{1}p_{1}^{T}+t_{2}p_{2}^{T}+t_{3}p_{3}^{T}+\ldots +t_{n}p_{n}^{T} \end{array}$$

In Formula (2), *t*, the score vector; *p*, the loading vector.

In this study, PCA was generally used to eliminate abnormal data outside the 95% confidence interval to improve the accuracy of qualitative analysis. The score could be used to explain the relationship and change trend between different samples, and the loading was applied to explain the spectral information of the data (27). Averaged spectra at different temperatures were analyzed by PCA, and then scores and loadings were introduced to find outliers and characterized peaks.

#### Continuous Wavelet Transform Analysis

Continuous wavelet transform (CWT) decomposes the signal into a series of superpositions of wavelet functions, obtained by translation and expansion. It also has the characteristics of a “mathematical microscope” (26). Wavelet is controlled by scaling parameters and translation parameters, and commonly used wavelet functions such as Daubechies, Coiflet, Symmlet, etc., (28). “Sym4” was selected to process the NIR spectra, improve the resolution of spectra, eliminate the background effect, and investigate the change of the protein secondary structure content during the heating process.

#### Two-Dimensional Correlation Spectroscopy Analysis

Two-dimensional correlation spectroscopy (2D-COS) is often used to analyze the dynamic characteristic spectra changes after the system is affected by external disturbance factors, and the changes are often expressed by Formulas (3–5) (29):

(3) $$\bar{y}\left(\nu ,t\right)=\{\begin{array}{c} y\left(\nu ,t\right)-\bar{y}\left(\nu \right)\;T_{min}\leq t\leq T_{max}\\ 0\text{ others} \end{array}$$

(4) $$\begin{array}{l} \bar{y}\left(v\right)=1/\left(T_{max}-T_{min}\right)\int_{T_{min}}^{T_{max}}y\left(\nu ,t\right)dt \end{array}$$

(5) $$\begin{array}{l} X\left(\nu_{1},\nu_{2}\right)=\Phi \left(\nu_{1},\nu_{2}\right)+i\text{Ψ}\left(\nu_{1},\nu_{2}\right) \end{array}$$

In Formulas (3–5), y(ν, t), the spectral intensity; ν, the wavelength; t, variable factor; X(ν, t), the spectral intensity at ν_1_ and ν_2_ under variable disturbance; Φ(ν_1_,ν_2_), synchronous spectra; Ψ(ν_1_, ν_2_), asynchronous spectra.

Spectral data are displayed with Φ(ν_1_, ν_2_) and Ψ(ν_1_, ν_2_). The synchronous 2D-COS indicates the degree of synergy between two dynamic spectral signals, and asynchronous 2D-COS shows the order of changes in the two spectral dynamic signals (30). Using the 2D-COS, the spectral dynamic changes of the system in this study under temperature disturbance could be analyzed, and the changes in the structure of the protein could be elucidated.

## Results and Discussions

### Protein Concentration Determination

**Figure 2:**
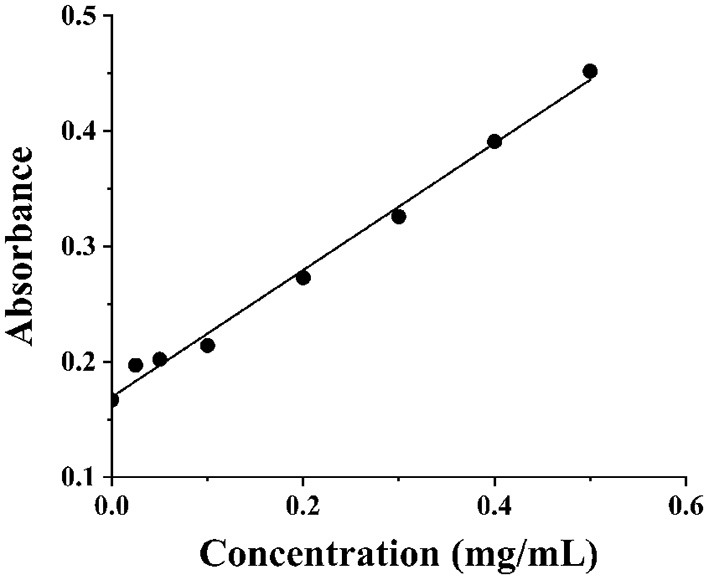
Protein standard curve.

The Ara h1 concentration was determined and the results were shown in Figure 2. It could be concluded that a good linear standard curve (protein standard curve y = 0.5504x + 0.1694, R^2^ = 0.9932) was established. Then Ara h1 solution was diluted 20 times, and the concentration was calculated as 5.16 mg·ml^−1^ according to the measured absorbance value. Then the solution was used for NIR spectra collecting.

### Near-Infrared Spectral Analysis of Ara h1 at Different Temperatures

#### Original Spectral Analysis of Ara h1

**Figure 3:**
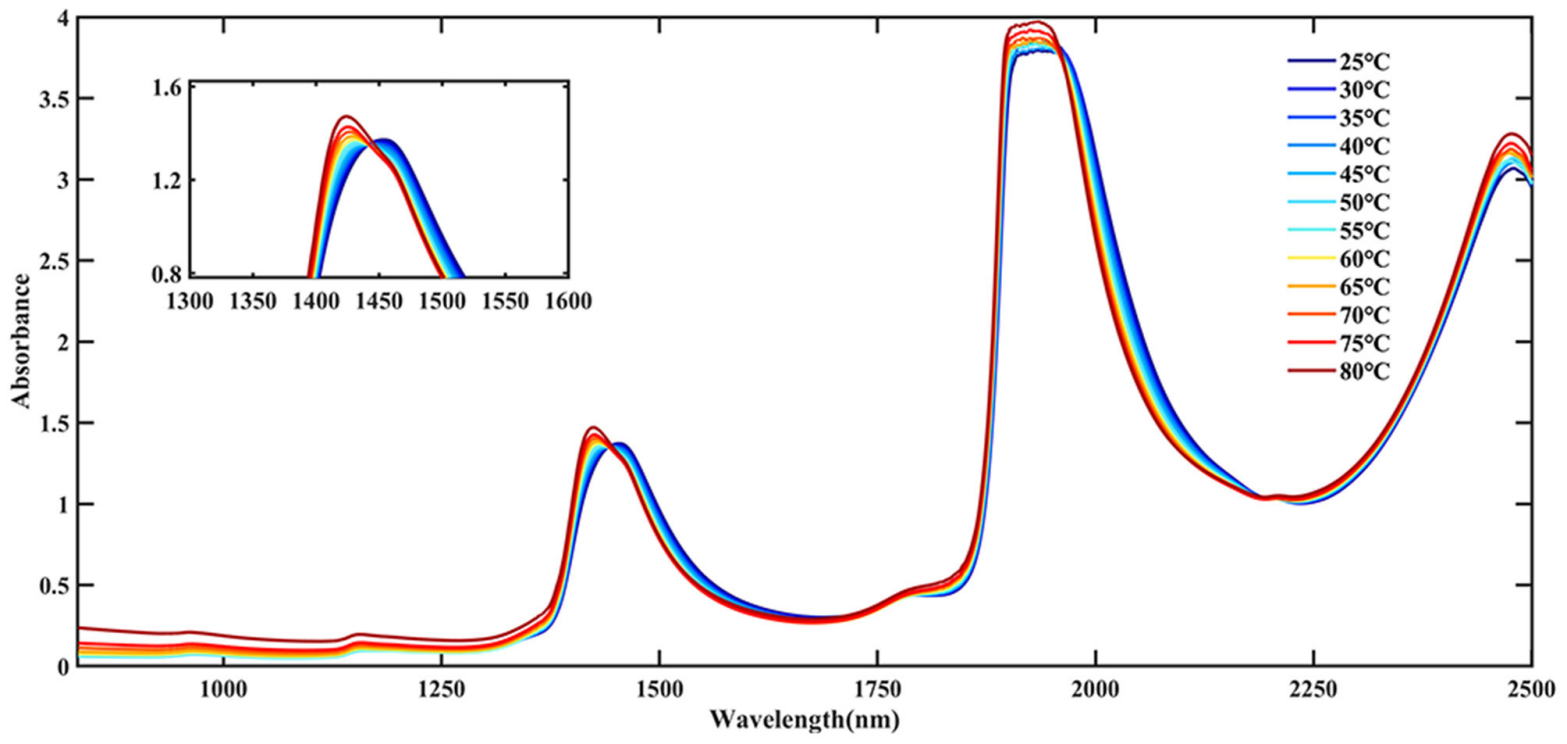
Near-infrared (NIR) spectra of Ara h1 aqueous solution at different temperatures.

Figure 3 showed the original NIR spectral of Ara h1 aqueous solution ranging from 25 to 80°C. It could be found that there were two broad peaks around 1,450–1,930 nm, respectively. The peak around 1,930 nm indicated that saturation occurred due to solvent absorption, and this band was not considered in the subsequent analysis. The broad peak near 1,450 nm was mainly the combined frequency absorption of the water O-H bending vibration and antisymmetric stretching vibration (15). During the heating process, a clear blue shift could be observed at the peak around 1,450 nm. That was, it moved from 1,454 to 1,425 nm, and an isothermal point was formed near 1,441 nm. It showed that the structure of the water solvation shell in the protein changed with the heating process, which affected the structure of the Ara h1 allergen protein.

#### Ara h1 PCA

**Figure 4:**
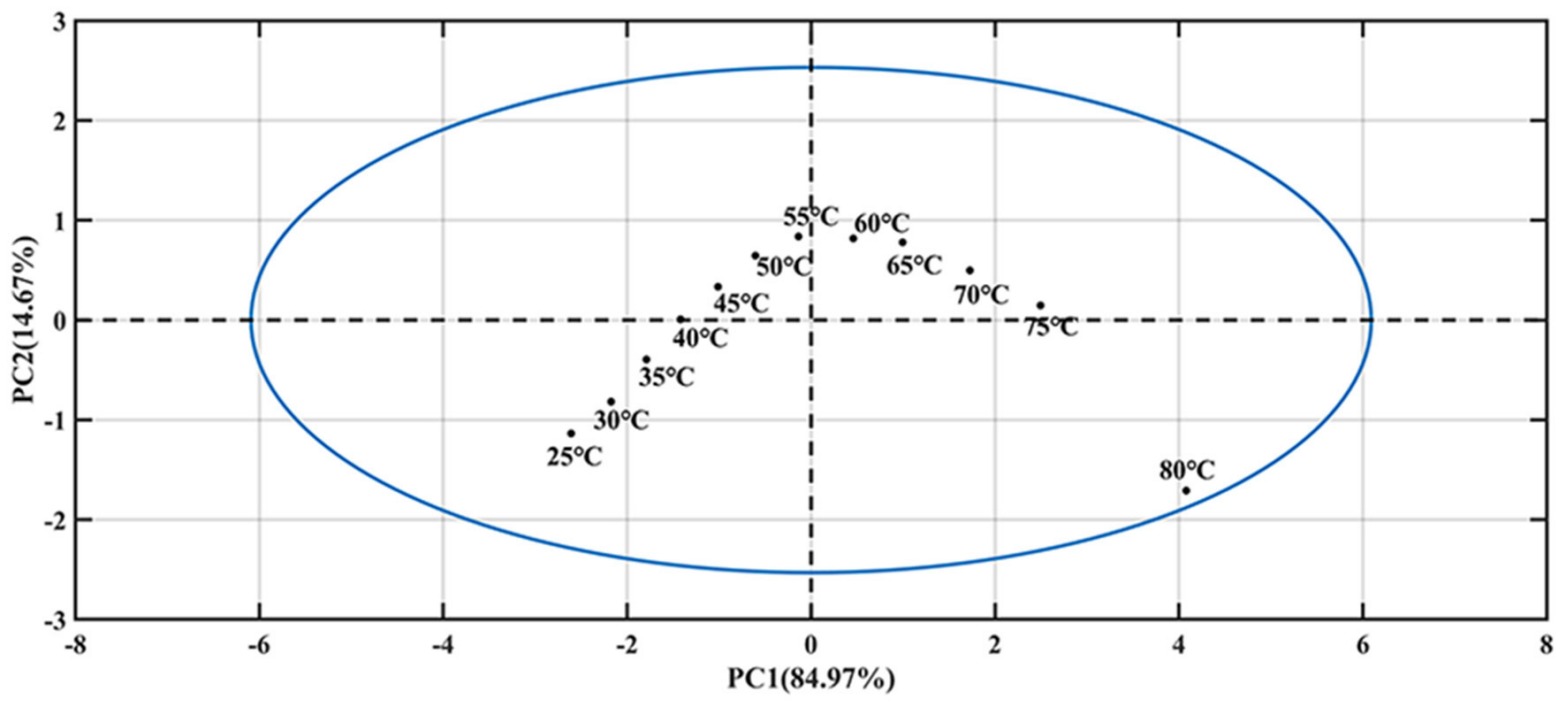
The principal component score of Ara h1 aqueous solution at different temperatures.

The first principal component (PC 1) explains the largest change in the data and contains the most information. Therefore, in this study, the temperature was the only external interference factor, so it was summarized in the PC 1. The change of protein structure due to temperature interference was the second important influencing factor, so it was summarized in the second principal component (PC 2). PCA was introduced to find more specific information and the results were shown in Figure 4. It was indicated that the PC 1 contributed an 84.97% variance of the whole spectra. The PC 2 contribution was 14.67%, which should be caused by the structural changes of Ara h1 during the heating process. There was a mutation point around between 55 and 60°C. It showed that the structure of Ara h1 changed significantly at this temperature, which might affect its sensitization ability. Combining with the original spectra that showed baseline drifted around 80°C, a small amount of flocculent precipitation was generated in the sample cell under this temperature. Therefore, the spectra were precluded in the subsequent analysis at 80°C.

**Figure 5:**
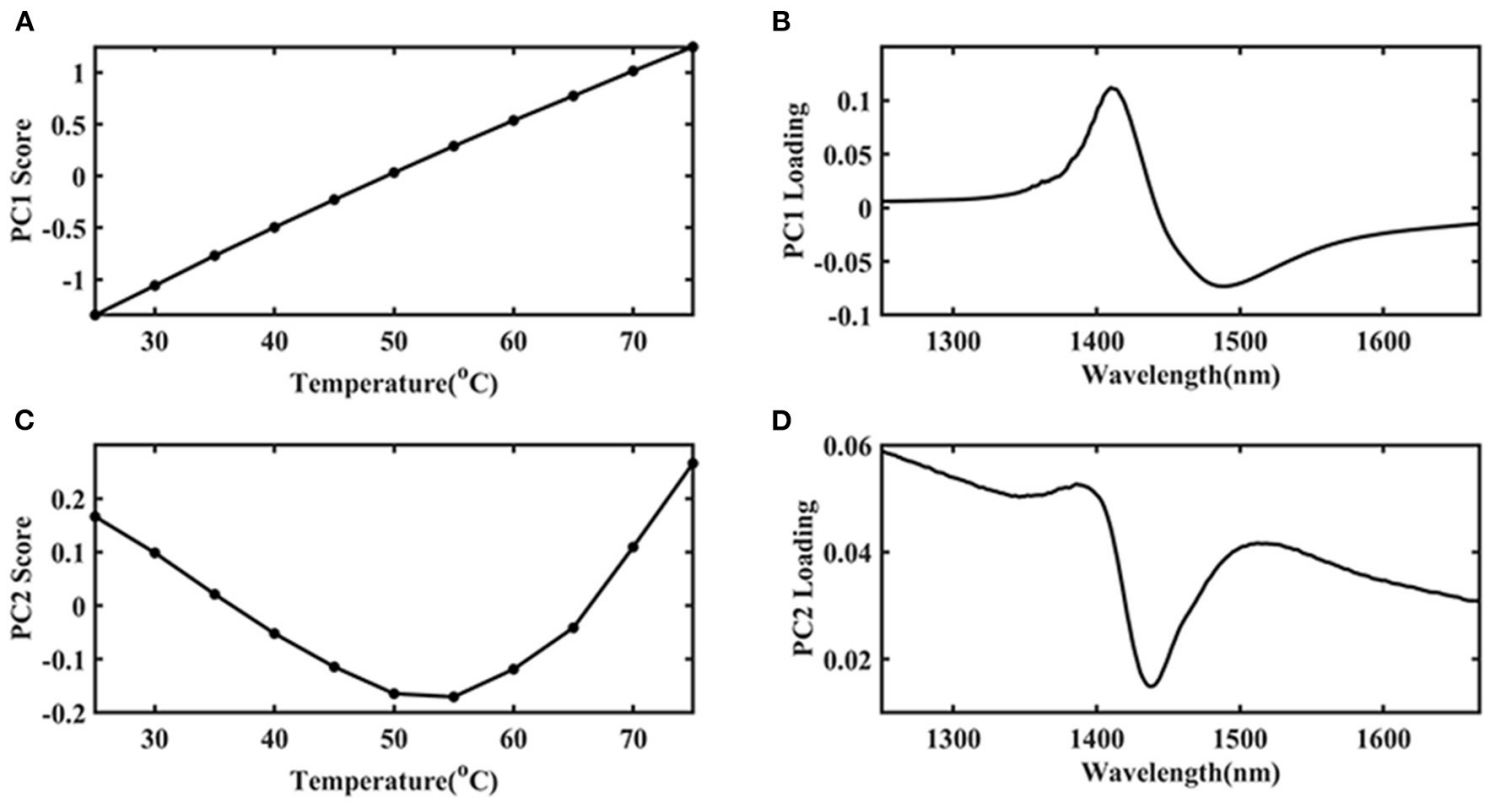
The first principal component (PC 1) score plot of Ara h1 aqueous solution **(A)** and its loading plot **(B)** at different temperatures, the second principal component (PC 2) score plot of Ara h1 aqueous solution **(C)**, and its loading plot **(D)** at different temperatures. (1,250–1,667 nm).

Due to the high absorption of water, the structure information of Ara h1 based on different temperatures was difficult to analyze. In this study, water was applied as a probe to investigate the effect of temperature on water structure, which could reflect the impact of temperature on Ara h1 structure indirectly based on aquaphotomics. PCA was performed on the spectra in the range from 1,250 to 1,667 nm, which was assigned as the water O-H overtone band, and the score and loading plots were shown in Figure 5. It was indicated that the PC 1 score (Figure 5A) gradually increased as the temperature increased, and the trend showed an excellent linear relationship (linear fitting: R^2^ = 0.9987) which proved that temperature had a significant impact on the Ara h1 aqueous solution. Combined with the loading plot (Figure 5B), it was found that a positive peak and a negative peak appeared at 1,414 and 1,489 nm, which could be attributed to the non-hydrogen bond absorption peak in free water and the strong hydrogen bond absorption peak of four hydrogen bonds in water molecules. The structure of the Ara h1 aqueous solution had an obvious inflection point at about 55°C from the PC 2 plot in Figure 5C. It indicated that its structure had undergone a major change, this change might be caused by the negative peak at 1,440 nm (Figure 5D). The total absorbance was decreasing at 1,440 nm with temperature increasing, forming a negative peak, which could be attributed to a weak hydrogen bond absorption peak in the water molecules. The weak and strong hydrogen bonds in the water molecules were constantly being destroyed, and the non-hydrogen bonding structure in free water was constantly increased, leading to changes in the structure of water molecules, free water was increased, and hydration was weakened, which affected the water solvation shell interaction between Ara h1 and water. The hydrophobic effect was enhanced, and the allergenicity of the protein might also be reduced.

#### Continuous Wavelet Transform Analysis of Ara h1

**Figure 6:**
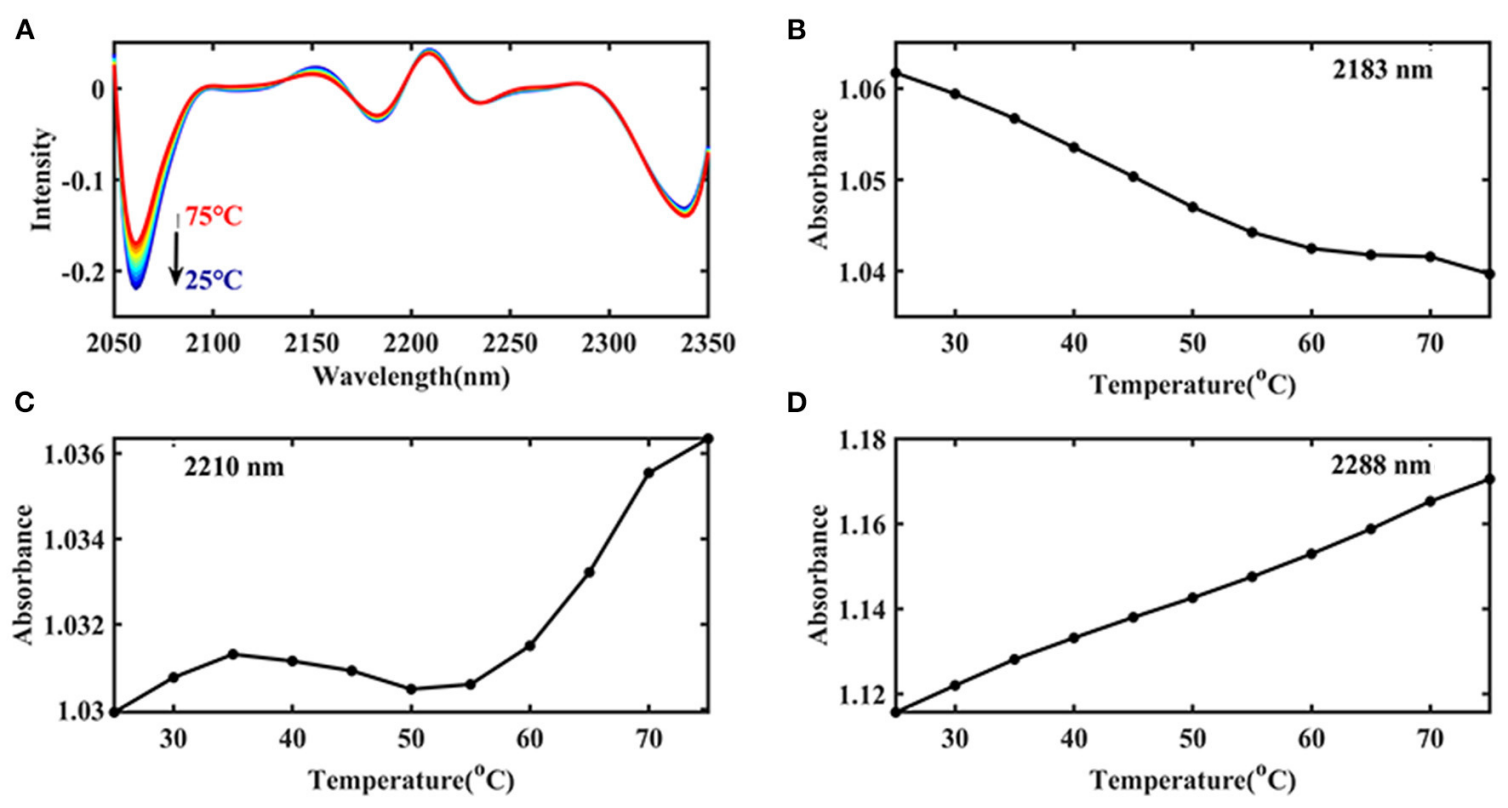
Transformed spectra calculated by continuous wavelet transform (CWT) of Ara h1 aqueous solution (2,050–2,350 nm) **(A)**; Absorbance of the peaks at 2,183 nm **(B)**, 2,210 nm **(C)**, and 2,288 nm **(D)** in the original spectra of Ara h1 aqueous solution during the heating process.

Then the spectra were handled by CWT in the wavelength range of 2,050–2,350 nm to find more information about the Ara h1, and “sym4” was selected as the wavelet base. Three negative peaks as shown in Figure 6A could be identified at 2,060, 2,183, and 2,342 nm, respectively. Combining with the characteristic absorption of protein (26, 30, 31) (Table 1), these peaks probably came from overlapping absorption of the N-H bending vibration of Ara h1 and the second overtone of an -OH bending vibration of water, combined frequency absorption of the C=O stretching vibration and the amide B/II, and -CH2 side-chain absorption, respectively. Two positive peaks appeared at 2,210 and 2,288 nm, which came from the absorption of β-sheet in the protein amide A/III bands and α-helical, respectively. Among them, the spectral change at 2,060 nm was more obvious, because the water absorption was stronger in the Ara h1 aqueous solution, and absorption of the second overtone of an -OH bending vibration in the water decreased significantly due to the heating effect, which masked the changes of the protein itself. Therefore, it is necessary to analyze the changes in protein structure in combination with aquaphotomics.

**Table 1:** Characteristic absorption of Ara h1 aqueous solution in near-infrared (NIR) spectra (26, 30, 31).

**Number**	**Wavelength (nm)**	**characteristic absorption band**
1	2,060	the N-H bending vibration; the second overtone of an -OH bending vibration of water
2	2,183	Amide B/II
3	2,210	β-fold
4	2,288	α- helix
5	2,342	-CH_2_ side chain

Figure 6B showed that the combined frequency absorption of C=O stretching vibration and amide B/II decreased significantly before 55°C, and after that, the absorption decreased slowly. The amide structures in the protein were destroyed due to the heating, and the C=O chain scission occurred. Figure 6C showed the content of β-sheet of Ara h1 increased from 25 to 35°C, the content began to decrease slightly from 35 to 55°C, and that, content rose relatively rapidly. The results showed that the structures of Ara h1 had undergone major changes at around 55°C, causing a rapid increase in the β-sheet content of the amide A /III. The α-helical content had been rising during the heating process (Figure 6D). The secondary structures of the protein changed, and the side chain structures of the protein were broken, resulting in a corresponding decrease in the sensitization ability of Ara h1.

#### Ara h1 2D-COS Analysis

**Figure 7:**
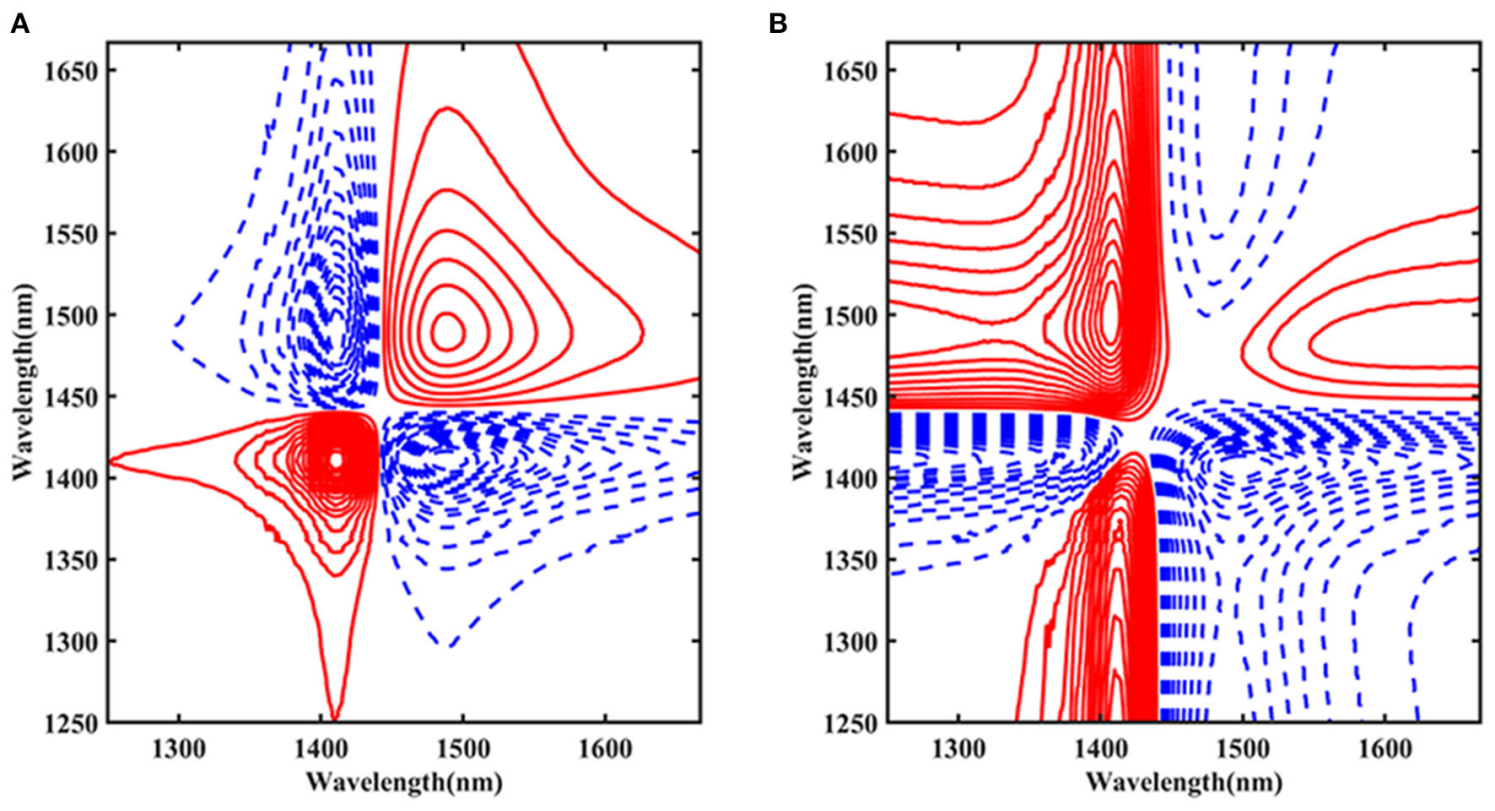
Two-dimensional correlation synchronous spectra of Ara h1 aqueous solution **(A)**; Two-dimensional correlation asynchronous spectra of Ara h1 aqueous solution **(B)**.

To further examine the influence of temperature on the structure of Ara h1 solution, the spectra were processed by 2D-COS from 1,250 to 1,667 nm (Figure 7). The red solid line indicated that the peak value was positive, and the blue dashed line indicated that the peak value was negative. Figure 7A showed the synchronization spectra and autocorrelation peaks at 1,414 and 1,489 nm were identified, respectively. The peak at 1,414 nm was primarily the non-hydrogen bond absorption. While the position at 1,489 nm mainly came from the strong hydrogen bond absorption with four hydrogen bonds in water molecules. Combined with the results of the asynchronous spectra (Figure 7B), we could conclude that the change of the absorption peak at 1,489 nm was earlier than that of 1,414 nm. During the heating process, the strong hydrogen bond structure of the water molecules in the Ara h1 solution was destroyed first, and the hydrogen bond network of the water-bound gradually disintegrated, the hydration was weakened, and the non-hydrogen bond free water structure in the system gradually increased. Under the impact of temperature, the hydrophobicity of protein-enhanced and precipitation was formed by dehydration. It could also account for the existence of a small number of flocculent precipitates in the experimental samples at 80°C.

#### Ara h1 Aquagram Analysis

**Figure 8:**
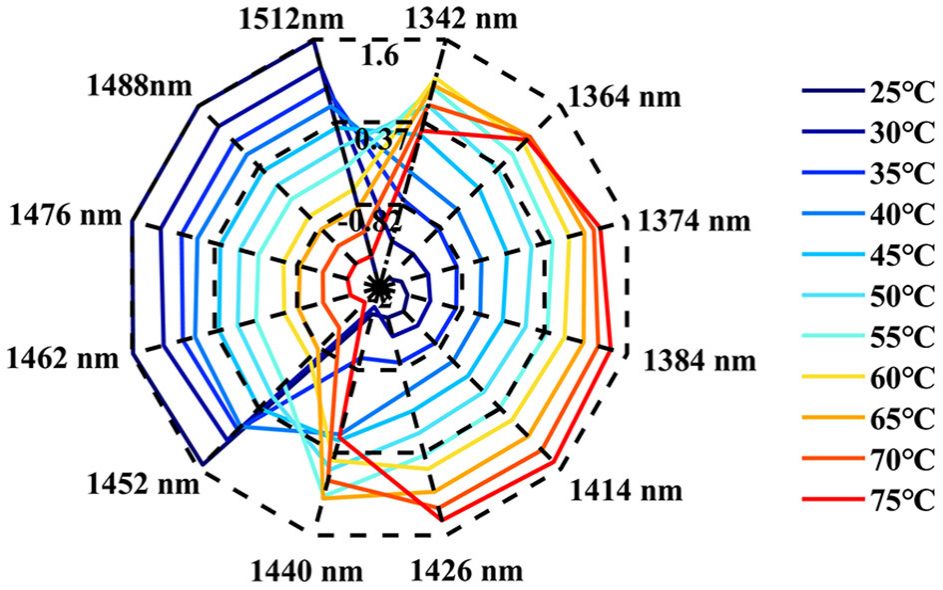
The aquagram of Ara h1.

The influence of temperature on the structure of Ara h1 was characterized by an aquagram. The characteristic absorption bands of the Ara h1 aqueous solution during temperature change were shown in Table 2 (32–39). The aquagram with the 12 WAMACS was shown in Figure 8. It showed that during the heating process, the water spectra of the Ara h1 aqueous solution gradually shifted from high wavelength to low wavelength, the structures of Ara h1 and its spectra were changed under the influence of temperature. The absorbance increased first, and then it decreased significantly when the temperature rose to 50°C at 1,440 nm. Therefore, combining with Table 2, the strong hydrogen bond was gradually destroyed by the increase of temperature, the weak hydrogen bond and other structures were formed. Under the influence of this effect, the water molecules were bounded by weak hydrogen bonds increasing. As the temperature rose again, the weak hydrogen bonds were also destroyed, the absorbance was weakened there, and the free water structure was formed. When the temperature was below 55°C, the WAMACS of the aquagram were mainly biased toward high wavelengths. Combined with Table 2, at this time, the hydrogen bond network structure of Ara h1 aqueous solution was stable, and the hydration was strong between water and the protein surface, which had little effect on the protein structure. When the temperature was higher than 55°C, the aquagram tended to be at low wavelengths, the hydrogen bond network was broken, hydration was weakened, the structure of Ara h1 aqueous solution was greatly changed, and the protein precipitated and aggregated, and the hydrophobicity of the protein increased. It led to the decrease of sensitization ability.

**Table 2:** Water matrix coordinates: characteristic absorption of water in NIR spectra (32–39).

**Number**	**Wavelength(nm)**	**Characteristic water absorption band**
1	1,342	V_3_
2	1,364	-OH-(H_2_O)_n, n, n = 1, 2, 4_: water solvation shell
3	1,374	V_1_ + V_3_
4	1,384	OH-(H_2_O)_n, n = 1, 4_: water solvation shell
		O_2_-(H_2_O)_4_: hydrated superoxide clusters
5	1,414	Non-hydrogen bonds for free water
6	1,426	OH Bend OH…O, hydration band
7	1,440	Water molecules with 1 hydrogen bond
8	1,452	Water solvation shell
9	1,462	Water molecules with 2 hydrogen bond
10	1,476	Water molecules with 3 hydrogen bond
11	1,488	Water molecules with 4 hydrogen bond
12	1,512	Strongly bound water; (V_1_, V_2_)

*V_1_, H_2_O symmetrical stretching vibration; V_2_, H_2_O bending stretching vibration; V_3_, H_2_O asymmetric stretching vibration*.

Combining the changes in the content of the protein secondary structure in the wavelength range of 2,050–2,350 nm, the analysis showed that the hydration was weakened during the heating process between the protein and the solvent water, and the Ara h1 aqueous solution precipitated and aggregated. The ordered structure was transformed to disorder, the binding site of the protein might be changed, which would affect the sensitization ability of peanut protein. This proved that using this non-labeled water as a probe combined with NIR spectroscopy could effectively characterize the structural changes of peanut allergens.

## Conclusion

Near-infrared spectral analysis technology and aquaphotomics were introduced into this research to explore the structural changes of the peanut allergen protein Ara h1 at 25–80°C. It was speculated that at the about 55°C mutation point, the binding site of the protein had undergone major changes under the influence of temperature. When the temperature rose to about 55°C, the protein structure was gradually destroyed, the content of the β-sheet began to rise, the hydrophobicity of the protein increased, and its sensitization ability decreased. It formed Ara h1 hypoallergenic protein, which could be used to treat peanut allergy.

A method for characterizing the regularity of protein structural changes without labeling was established through this research. Compared with the conventional analysis method for structural changes, it was easy to operate and had high sensitivity. At the same time, it was based on the interaction between the allergen protein Ara h1 and the water structure, revealed the temperature point of the Ara h1 protein structural changes. It laid a theoretical foundation for food processing technology and also provided a new idea to explore the interaction of various molecules in the life system.

## Data Availability Statement

The raw data supporting the conclusions of this article will be made available by the authors, without undue reservation.

## Author Contributions

MZ is the experimental designer and executor of this study. LL is mainly responsible for the preparation of peanut allergen Ara h1. CY, ZS, and XX are responsible for the data collection. LL is responsible for the conceptualization, funding, and supervision. HZ is responsible for the supervision and funding. All authors contributed to the article and approved the submitted version.

## Conflict of Interest

The authors declare that the research was conducted in the absence of any commercial or financial relationships that could be construed as a potential conflict of interest.
